# The complete mitochondrial genome of *Balanus trigonus* (Thecostraca, Balanomorpha, Balanidae) from South Korea

**DOI:** 10.1080/23802359.2021.1966335

**Published:** 2021-08-24

**Authors:** Seongjun Bae, Philjae Kim, Chang-Ho Yi

**Affiliations:** a Department of Ecology and Conservation, Marine Biodiversity Institute of Korea, Seocheon, Republic of Korea; b Department of Ocean Environmental Sciences, College of Natural Science, Chungnam National University, Daejeon, Republic of Korea; c Division of Ecological Conservation, Bureau of Ecological Research, National Institute of Ecology, Seocheon, Republic of Korea

**Keywords:** Balanidae, mitogenome, *Balanus trigonus*, phylogeny

## Abstract

The complete sequence of the mitochondrial genome of *Balanus trigonus* Darwin, 1854 was examined using next-generation sequencing analysis. The complete mitogenome of *B*. *trigonus* has 15,336 bp in length and comprises 37 genes, namely, 13 protein-coding genes (PCGs), 22 tRNAs, and two rRNAs. Both the gene order and characteristics are consistent with those of other species within the family Balanidae. Phylogenetic analysis based on complete mitogenomes revealed taxonomic relationships among members of the family Balanidae.

Barnacles of the genus *Balanus* Costa, 1778, within the family Balanidae in the order Balanomorpha, have a worldwide distribution that encompasses temperate and subtropical areas. To date, a total of 88 *Balanus* species have been described globally, of which 10 species have been reported from Korea (Kim 2011; Kim et al. 2020). Currently, however, the entire mitochondrial genome of only one (*Balanus balanus*) of these 10 species has been sequenced (Shen et al. 2014). Herein, we report the complete mitogenome of a further species, *Balanus trigonus* (MZ049958), which will contribute to assessments of the evolutionary relationships among barnacles.

Specimen of *B. trigonus* was collected in Tongyeong, Korea (34°49′41.8″N, 128°26′06.7″E) on 17 January 2021, at depths of between 0 and 1 m, and was subsequently identified based on morphological studies (Kim 2011; Kim et al. 2019). The voucher specimen (MABIK CR00248069) has been deposited in a deep freezer (−80 °C) of the National Marine Biodiversity Institute of Korea (Seongjun Bae, silverto@naver.com, Seocheon, Korea). Total genomic DNA was extracted from the specimen using a DNeasy Blood & Tissue DNA kit (Qiagen, Hilden, Germany), from which a genomic library was constructed using a QIAseq FX single-cell DNA library kit (Qiagen, Hilden, Germany) using paired-end reading. Next-generation sequencing analysis was conducted using an Illumina HiSeq 4000 system (Illumina Inc., USA). The complete mitogenome was reconstructed using Geneious Prime 2020.11.0 (Biomatters Ltd, Auckland, New Zealand).

The mitogenome of *B. trigonus* is 15,336 bp in length and consists of 13 protein-coding genes (PCGs), 22 transfer RNA (tRNA) genes, and two ribosomal RNA (rRNA) genes. The overall nucleotide composition is 37.1% A, 15.6% C, 11.6% G, and 35.7% T. A majority of the PCG start codons are ATG (COX2, COX3, CYTB, ND2, ND5, and ND6), whereas the ATP6, ND1, and ND4 genes start with ATA, and ATT is the start codon for ATP8 and ND3. Exceptionally, AAA and GTG are used as alternative start codons for COX1 and ND4L, respectively. Notably, *B*. *balanus* (KM660676) similarly uses AAA as an alternative start codon for COX1. The most common stop codon is TAA (ATP6, ATP8, COX1, ND1, ND2, ND4L, ND5, and ND6), followed by TAG (CYTB). The remaining four PCGs (COX2, COX3, ND3, and ND4) were found to have an incomplete stop codon ‘T––’.

The dataset used for phylogenetic analysis included the 13 PCGs from 28 species in the 12 families and those from the three barnacles *Lepas australis* (NC_025295), *Lepas anserifera* (NC_026576) and *Glyptelasma annandalei* (MH891848) were used as outgroups. The best-fit substitution was estimated using jModelTest 2.1.1 (Guindon and Gascuel 2003; Darriba et al. 2012). Maximum likelihood (ML) analysis was conducted using PhyML 3.1, based on the TVM + I + G model with 1,000 replications of bootstrap assembly (Guindon et al. 2010). In the ML tree thus obtained, *B*. *trigonus* was found to cluster as a sister group with *Fistulonalanus albicostatus* (MK617531) and *Amphibalanus amphitrite* (KF588709), which are related species within the family Balanidae.

Previously considered to be a single monophyletic, Archaeobalanidae and Balanidae have been combined into one large family in a recent phylogeny study through molecular evidence (Tsang et al. 2017; Chan et al. 2021). In our results, *Armatobalanus alliusm* (presently in Balanidae) and Pyrgomatidae form a monophyletic clade (Figure 1). This pattern is consistent with those reported previously (Chan et al. 2021; Ji et al. 2021; Mao et al. 2021). However, Simon-Blecher et al. 2007 and Tsang et al. 2014 stated *A*. *allium* should be pyrgomatid barnacles based on morphology and molecular evidence. A revision of the taxonomic status of *A*. *allium* will be a further research direction.

**Figure 1.**
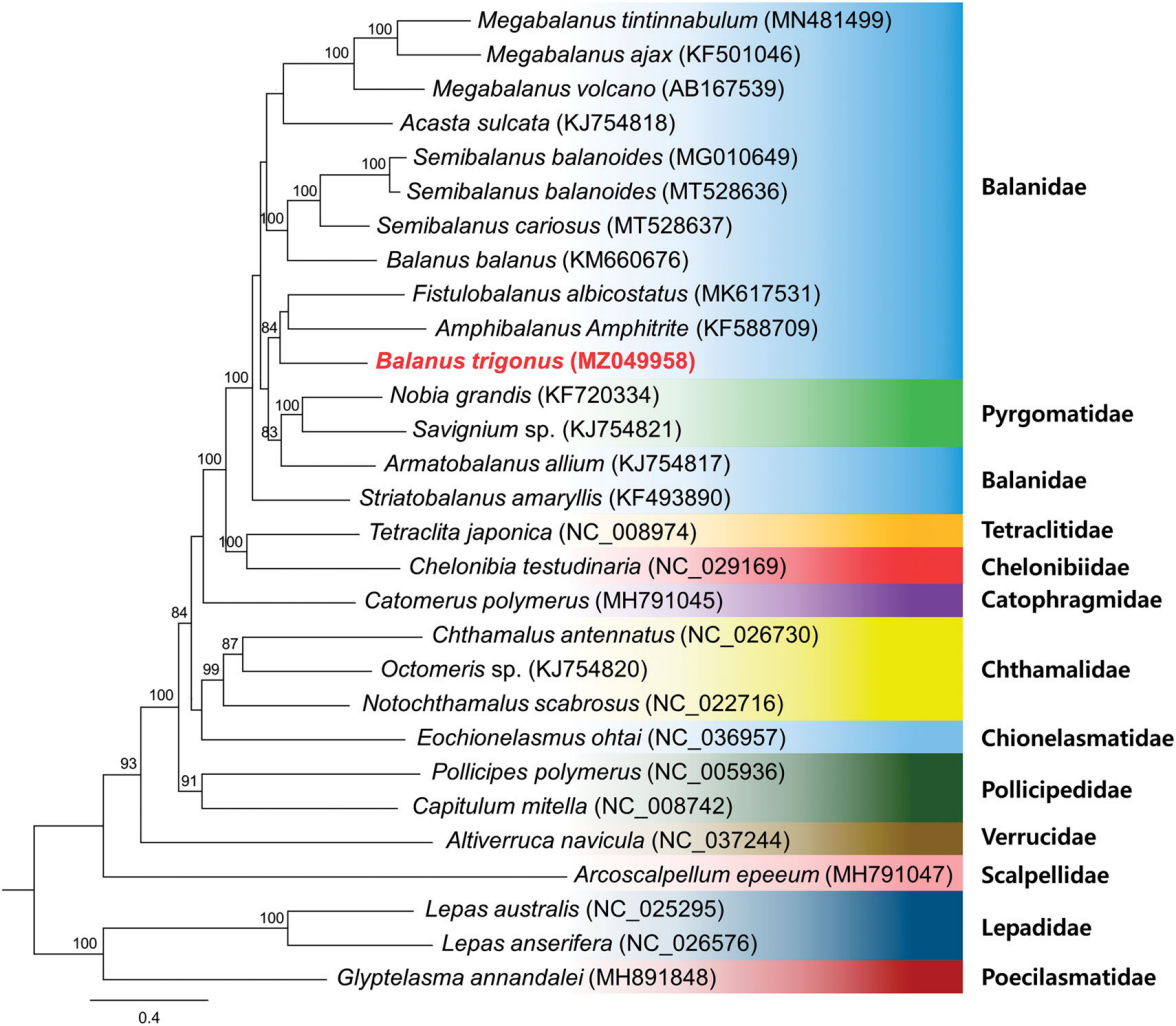
Phylogenetic tree inferred by maximum-likelihood using of 13 protein-coding genes of 28 barnacles mitochondrial genomes, including *B*. *trigonus* (MZ049958). Bootstrap support values based on 1,000 replicates are displayed on each node as >70.

We found the molecular phylogenetic relationships between Balanidae and Pyrgomatidae to be inconsistent with the current classification based on morphological features, thereby highlighting the necessity for further clarification of the phylogenetic classification between Balanidae and Pyrgomatidae, and the need for further research regarding morphological reclassification. In this regard, the mitogenome sequence obtained in the present study will serve as valuable a genomic resource, contributing to further molecular studies on the evolution of the family Balanidae.

## Data Availability

The genome sequence data that support the findings of this study are openly available in GenBank of NCBI at (https://www.ncbi.nlm.nih.gov/) under the accession no. MZ049958. The associated BioProject, SRA, and Bio-Sample numbers are PRJNA717899, SRR14270317, and SAMN18515342, respectively.
